# Application of a new percutaneous multi-function pedicle locator in minimally invasive spine surgery

**DOI:** 10.1038/s41598-021-01027-6

**Published:** 2021-11-02

**Authors:** Xiaojian Liu, Hairun Liu, Yushan Wang

**Affiliations:** 1 Department of Pharmacology, School of Basic Medical Sciences, Jinzhou Medical University, Jinzhou, 121001 Liaoning People’s Republic of China; 2 Department of Orthopedics, Jinzhou Central Hospital, Jinzhou, 121001 Liaoning People’s Republic of China; 3 Department of Orthopedics, Antai Hospital, No. 9~9-1, Xindalu, Section 2, Heping Road, Guta District, Jinzhou, 121001 Liaoning People’s Republic of China

**Keywords:** Spinal cord diseases, Techniques and instrumentation

## Abstract

In this study, a new percutaneous multi-function pedicle locator was designed for personalized three-dimensional positioning of a pedicle in minimally invasive spine surgery (MISS) without computer-assisted navigation technology. The proposed locator was used in a number of patients during MISS, and its advantages were analyzed. Based on the position of a pedicle determined by computed tomography (CT) and fluoroscopic images of a patient, 6 lines and 2 distances were used to determine the puncture point of a pedicle screw on skin, while 2 angles were used to indicate the direction of insertion of a pedicle guide needle from the patient's body surface. The results of the proposed locator were compared with those of the conventional freehand technique in MISS. The potential benefits of using the locator included enhanced surgical accuracy, reduced operation time, alleviation of the harmful intra-operative radiation exposure, lower costs, and shortened learning curve for young orthopedists.

## Introduction

In recent years, with the vigorous development of spinal endoscopy, the conventional open spinal surgery has been gradually replaced with minimally invasive spine surgery (MISS), and percutaneous pedicle screw fixation has markedly attracted surgeons’ attention^1,2^. The accurate positioning of the pedicle is crucial in both conventional open approach and MISS^3^. The angle of the pedicle can change due to the extroversion angle of the pedicle, tilted head or tail, and even possible lateral curvature and rotation caused by serious degeneration, which may disturb the accuracy of pedicle screw placement. This justifies why spinal surgery typically requires two-dimensional (2D) fluoroscopic imaging, and it is still not easy for the experienced orthopedic surgeons to ensure that screws can be placed accurately^4^. Challenges in spinal surgery are mainly available in the process of pedicle screw misplacement^5^. At present, the accuracy of pedicle screw placement can be ensured with navigated robot-assisted spinal surgery^2,3^. However, this method is extremely hindering its popularization. To date, however, the individualized placement of pedicle screws based on the precise measurement of computed tomography (CT) images has not yet been reported to be guided by specialized three-dimensional (3D) instruments in MISS.

In the present study, a simple and practical instrument has been designed for MISS. It could greatly improve the accuracy of screw placement, create channels in pedicle, shorten significantly the operation time, and decrease harmful intra-operative radiation exposure, which also reduced the cost of surgery. Moreover, it could assist young orthopedists to shorten the learning curve in the majorities of spinal surgeries. The percutaneous multi-function pedicle locator was clinically applied, and it achieved the predefined objectives.

## Materials and methods

### The basic principles of designing percutaneous multi-function pedicle locator

In the pedicle approach, a guide needle was inserted percutaneously into the pedicle in MISS. However, the percutaneous puncture point was not the vertical projection point of the pedicle on the skin. Because each pedicle has an extroversion angle and a head or tail tilt angle, the puncture point on skin should be on the lateral side and head or tail side of the pedicle projection point on the skin. However, there are individual differences in the angles of each pedicle of spine, especially the angles of the head or tail tilt often vary with the curvature of the spine, and the largest difference is found in the sacral spine. Thus, determining an appropriate point on skin to puncture and keeping the correct direction of a needle along the pedicle during insertion are crucial during surgery, which can be achieved by pre-calculation based on imaging and application of the percutaneous multi-function pedicle locator in surgery.

### Description of a novel percutaneous multi-function pedicle locator

**Figure 1:**
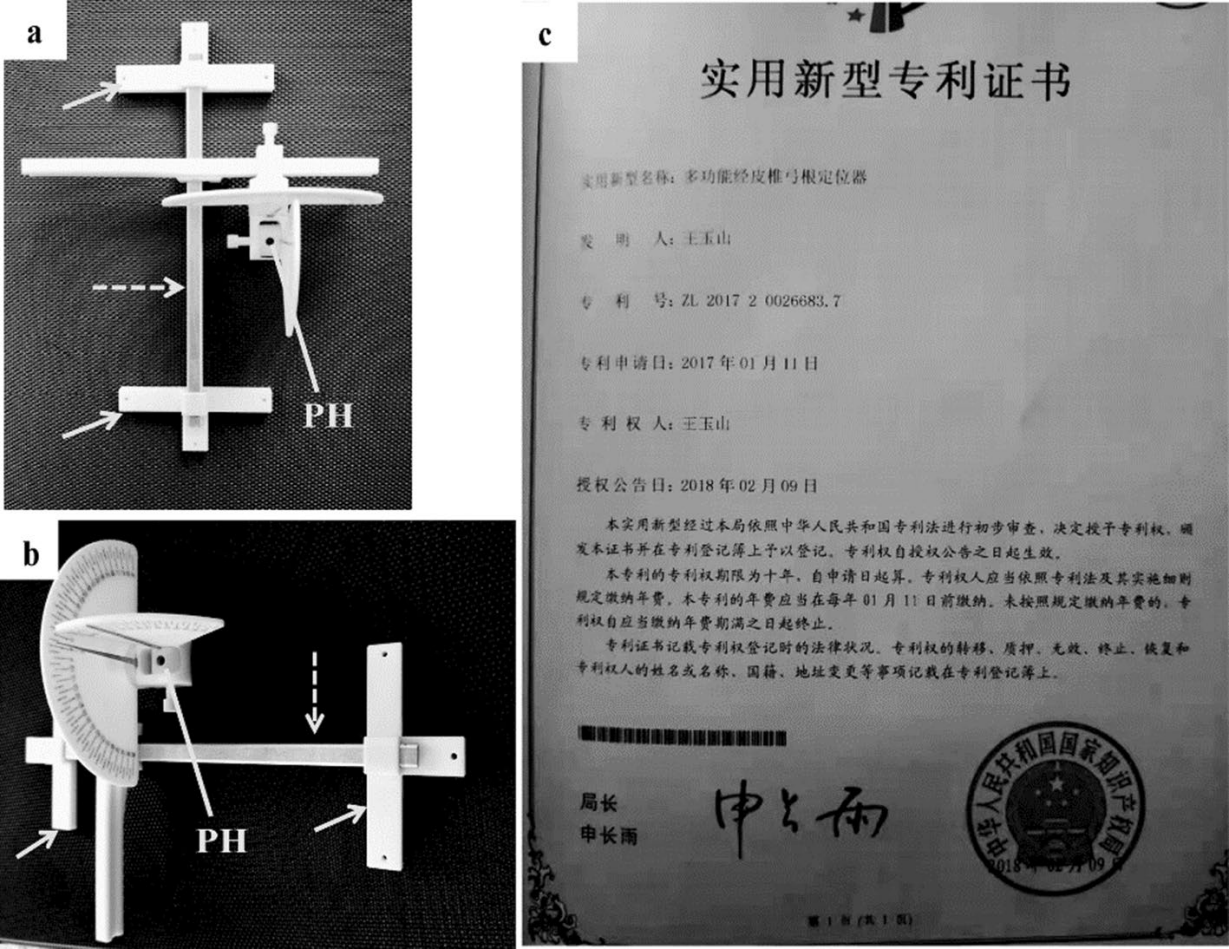
The proposed percutaneous multi-function pedicle locator. The structure of the locator was shown in (**a**) and (**b**), in which areas indicated by an arrow are the main parts (the base) of the locator, and areas indicated by a dotted arrow represent the longitudinal axis of the base; (**c**) The patent certificate of the proposed percutaneous multi-function pedicle locator. PH = A needle passing through the skin to the target point (pinhole surgery).

The structure of percutaneous multi-function pedicle locator is shown in Fig. 1a,b. The study was registered at The China National Intellectual Property Administration (Registeration No. ZL201720026683.7) (Fig. 1c). This locator was composed of base, longitudinal axis, horizontal and vertical angles, pinhole, and adjustable screws. There were rotatable hands on the two angles. Based on the anatomical features of the pedicle, the locator was designed to set two angles discretionarily. If the pinhole was regarded as a point, the line passing through the pinhole in the directions limited by the two angles was the one through which the needle entered the vertebral body at the posterior wall of the pedicle. The locator was manufactured by Beijing Fule Technology Development Co., Ltd. (Beijing, China). It could be produced as a sterile disposable medical device. The informed consent about application of the locator in MISS was obtained from all subjects. The protocol was approved by Ethics Committee of the Jinzhou Central Hospital (Jinzhou, China). All the surgical procedures were performed according to relevant institutional guidelines and national regulations.

### Application of the proposed percutaneous multi-function pedicle locator

The surgical procedures included percutaneous pedicle screw surgery, vertebroplasty, percutaneous vertebroplasty (PVP), and percutaneous kyphoplasty (PKP).

1. Principle

For the above-mentioned surgical procedures, a "bone channel" was created through the pedicle to enter the vertebral body. A "bone channel" which entered the vertebral body at the posterior wall of the pedicle was parallel to the upper and lower endplates of vertebral body. It was not interfered by changes of body posture, patient size, and spinal degeneration, but depended on the individual differences of each vertebral body and the purpose of surgery. Therefore, the same reference plane was selected for different vertebral bodies, and the mentioned channel was accurately created. A patient was mainly in the prone position in spinal surgery, so that the horizontal plane was selected as the reference plane. Concerning the correct application of the locator, it is very important to ensure that the base of locator was parallel to the ground level in surgery. Then, the vertical line of the locator’s base plane was considered as reference to set the angles of head and tail.

2. Determination of parameters based on X-ray and CT images before surgery.

According to the method of 622-1, one point (the percutaneous puncture point) was determined by 6 lines, 2 angles, and 2 distances.

**Figure 2:**
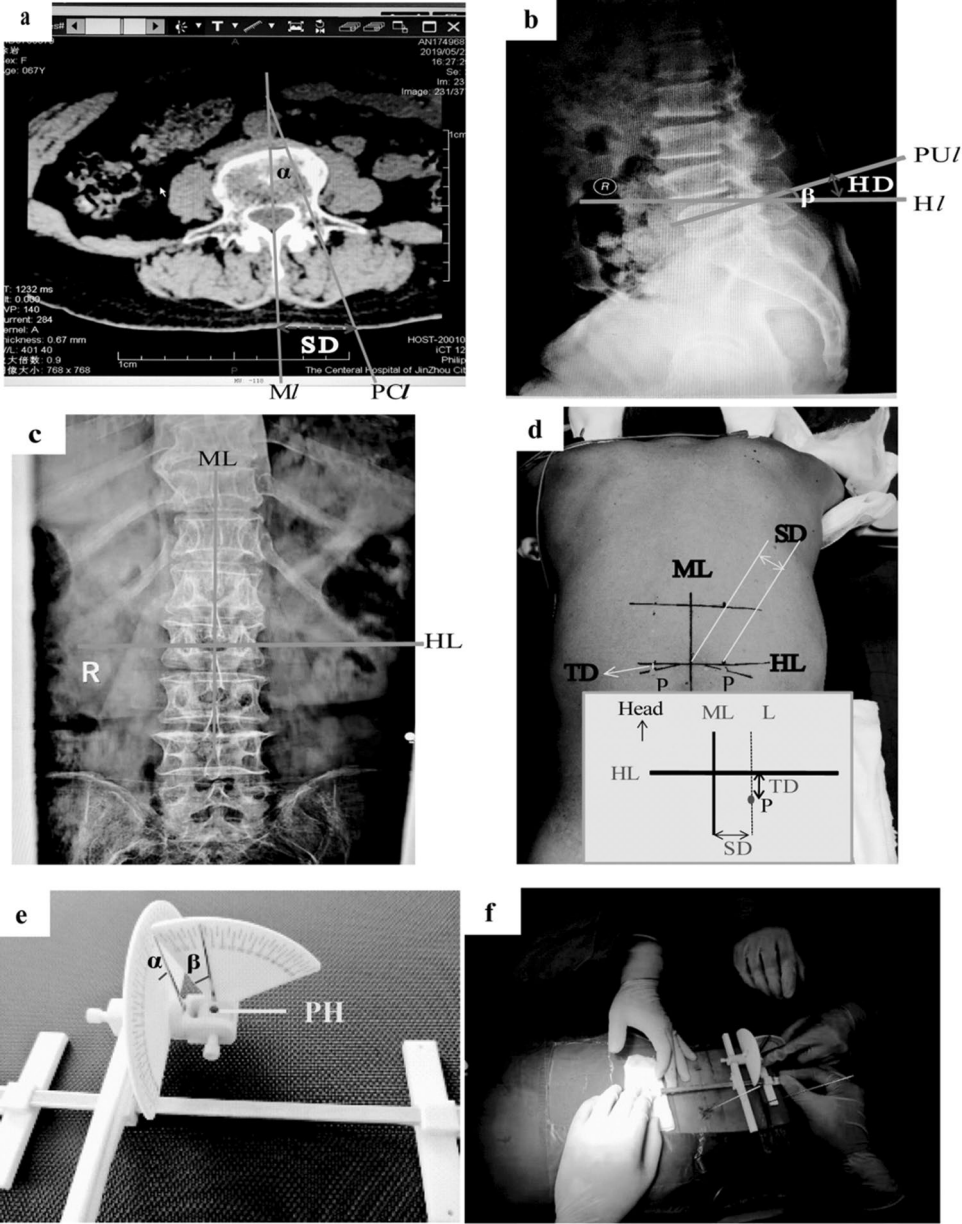
Four lines, two distances, and two angles presented for application of the locator in a surgery. Graph (**a**) show M*l* and PC*l* lines, SD, and angle α in a cross-sectional CT image; Graph (**b**) displays PU*l* and H*l* lines, H/TD, and angle β in a lateral X-ray image. Graph (**c**) illustrates ML and HL lines on the body surface using an X-ray image. Graph (**d**) shows ML and HL lines, SD and H/TD, and point P on the body surface. Graph (**e**) depicts angles α and β on the two scale dials of the locator. Graph (**f**) shows the practical utilization of the locator. M*l* and ML, linear median posterior; PC*l*, pedicle channel line; PU*l*, needle entry point on pedicle; H*l* and HL, horizontal lines; SD, side opening distance; H/TD, head or tail tilt distance; angle α, extroversion angle; angle β, head or tail tilt angle; P, percutaneous puncture point; PH, a needle passing through the skin to the target point (pinhole surgery).

There were ***4 lines*** based on images, including a linear median posterior (M*l*) line and a pedicle channel line (PC*l*) in a cross-sectional CT image (Fig. 2a), a line passing through the needle entry point on pedicle (PU*l*) that was parallel to the upper edge of vertebral body (terminal plate of vertebral body), and a horizontal line passing through the needle entry point on pedicle (H*l*). Both PU*l* and H*l* could be observed in a lateral X-ray image (Fig. 2b).

*The ****other 2 lines*** were marked on the body surface of the patient, including a linear median posterior line (**ML***,* both M*l* and ML were in the sagittal aspect of the body) and a horizontal line (**HL**, it was the same with H*l* in the lateral X-ray image) through the projection of bilateral pedicles on the body surface (it was marked in the X-ray fluoroscopic image) (Fig. 2c).

The ***2 angles*** included the extroversion angle of a pedicle (namely **angle α**), which included the angle between M*l* and PC*l* through the pedicle in the CT image (Fig. 2a) and the head or tail tilt angle (namely **angle β**) between PU*l* and H*l* in the lateral X-ray image (Fig. 2b).

The ***2 distances*** were side opening distance (SD) and the head or tail tilt distance (H/TD). SD in the CT image was the distance from the point where the line M*l* passed through skin to the point where the line PC*l* passed through skin. While on the patient’s body surface, SD was the distance from the percutaneous puncture point to the ML line (Fig. 2a). H/TD in the X-ray image was the distance from the point where the PU*l* line passed through skin to the point where the H*l* line passed through skin. However, on the patient’s body surface, H/TD was the distance from the percutaneous puncture point to the HL line (Fig. 2b).

The percutaneous puncture point “P” on the patient’s body surface was determined based on the above-mentioned "lines" and "distances" (Fig. 2d). The angles α and β were shown on the two scale dials of the proposed locator (Fig. 2e).

3. Steps required for practical application of the locator

After measurement of the above-mentioned "lines", "angles", and "distances", the locator was practically applied in a surgery as follows:

I. *Posture *The prone position was adopted, and the lateral position was considered for special needs.
II. *Mark ML and HL lines on the patient’s body surface *The ML line was marked according to the spinous process, and the HL line was marked through X-ray fluoroscopy and a level instrument.
III. *Mark the percutaneous puncture point “P” on the patient’s body surface according to SD and H/TD *A dashed line (L) that was parallel to ML was marked and SD (cm) away from ML was obtained. Then, point “P” above the HL line was marked on the L line, and HD (cm) away from HL (in case that pedicle was tilted to the head) was obtained. Or point “P” below the HL line was marked on the L line, and TD (cm) away from HL was obtained (in case that the pedicle was tilted to the tail). Further details are illustrated in Fig. 2d.

IV. *Determination of the direction of a surgical needle passing through the point “P” by setting the two angles on the locator* The position of the dials was adjusted manually to angle α and angle β, respectively, according to the CT image (angle α is shown in Fig. 1a) and the lateral X-ray image (angle β is shown in Fig. 2b). The locator was placed horizontally on the back of a patient by adjusting the screws at the bottom of the locator using a level instrument, and the longitudinal axis of the locator was overlapped or parallel to the ML line (Fig. 2e).
V. *Placement of a surgical needle in the pedicle* A surgical needle (and cannula) was placed into the pinhole (PH, Fig. 2e) of the locator according to the direction limited by angle α and angle β, and the distal end of the cannula was aligned to the point “P” on skin of the patient. More specifically, the line passing through the pinhole on the locator in the direction limited by the two angles should pass through the point “P” on skin, and then, this line had the same direction and position with the surgical needle in the pedicle. After that, a 1.5 mm titanium Kirschner wire was put into the cannula and inserted percutaneously into the dorsal bony cortex of the pedicle (Fig. 2f).
VI. *The steps of III–V were repeated until placement of all surgical needles*
VII. *Confirmation of accurate positioning of the surgical needles* X-rays were used to confirm whether all surgical needles were placed accurately. Further amendment was performed if necessary.
VIII. *Completion of the remaining surgical procedures.*

Important notes:

1. ML and HL lines were marked accurately on the patient’s body surface.
2. The longitudinal axis of the locator was overlapped or parallel with the ML line.
3. The locator cannot be tilted left or right.
4. If the patient had scoliosis, the camber angle was adjusted appropriately, so that the surgical needles of different segments were kept in a line.

### Statistical analysis

Statistical analyses were performed using the SPSS software 22.0 (IBM Corp., Armonk, NY, USA, https://www.ibm.com/cn-zh/analytics/academic-statistical-software). The numerical data were presented as mean ± standard deviation. The Student’s t-test was used to compare surgical parameters between the two groups. *P* < 0.05 was considered statistically significant.

### Consent for publication

All authors approved the final draft. No other consents are necessary for this study.

## Results

### Patients

**Figure 3:**
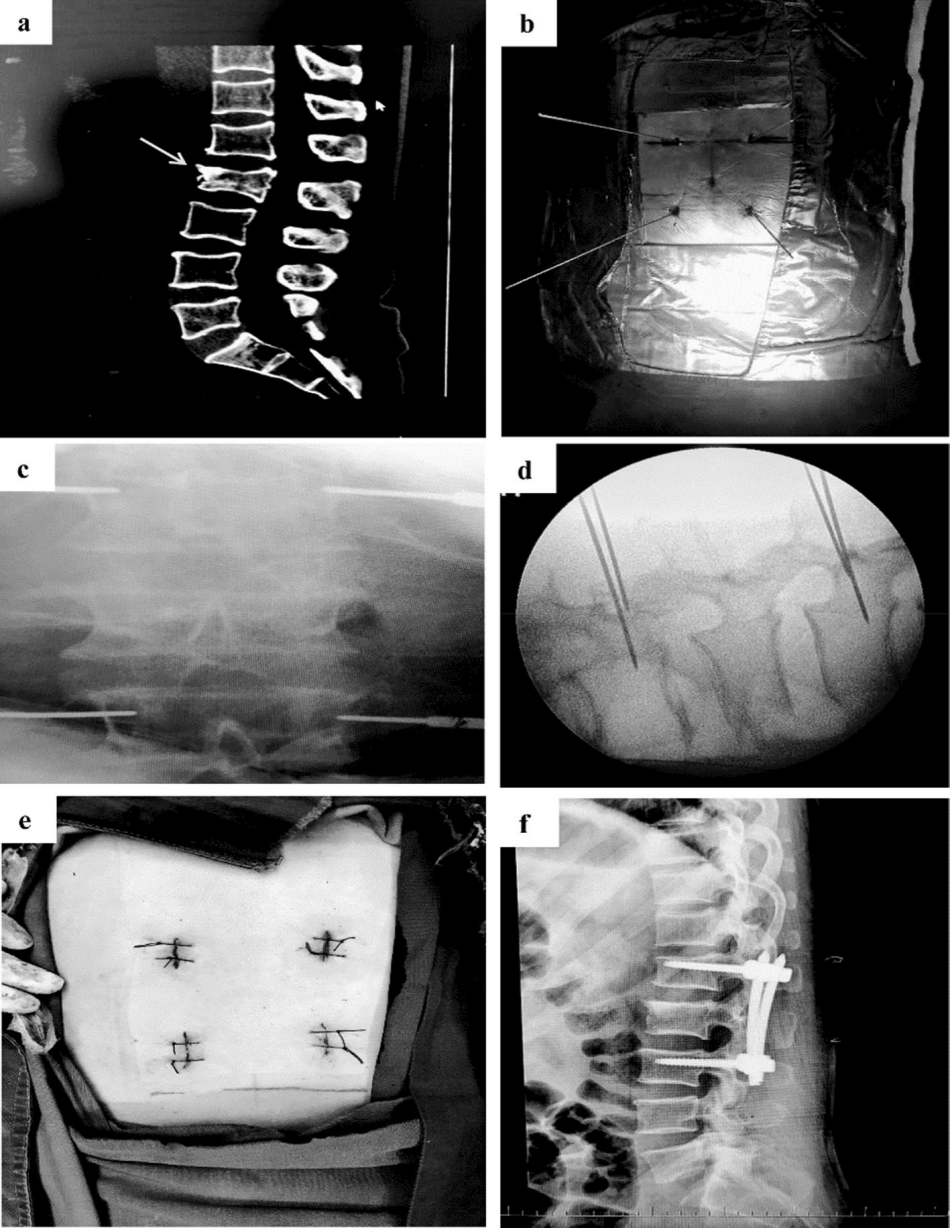
Reduction of vertebral compression fractures with percutaneous pedicle screw internal fixation using the proposed locator. Graph (**a**) shows a CT image of a vertebral compression fracture (lumbar #2). Graph (**b**) illustrates that the Kirschner wires were inserted into the pedicles of both sides of the lumbar #1 and #3 guided by the locator. Graphs (**c**) and (**d**) show that the accuracy of pedicle guide needle placement was confirmed by 2D fluoroscopy. Graph (**e**) displays the closed incisions. Graph (**f**) depicts DR image taken postoperatively.

**Figure 4:**
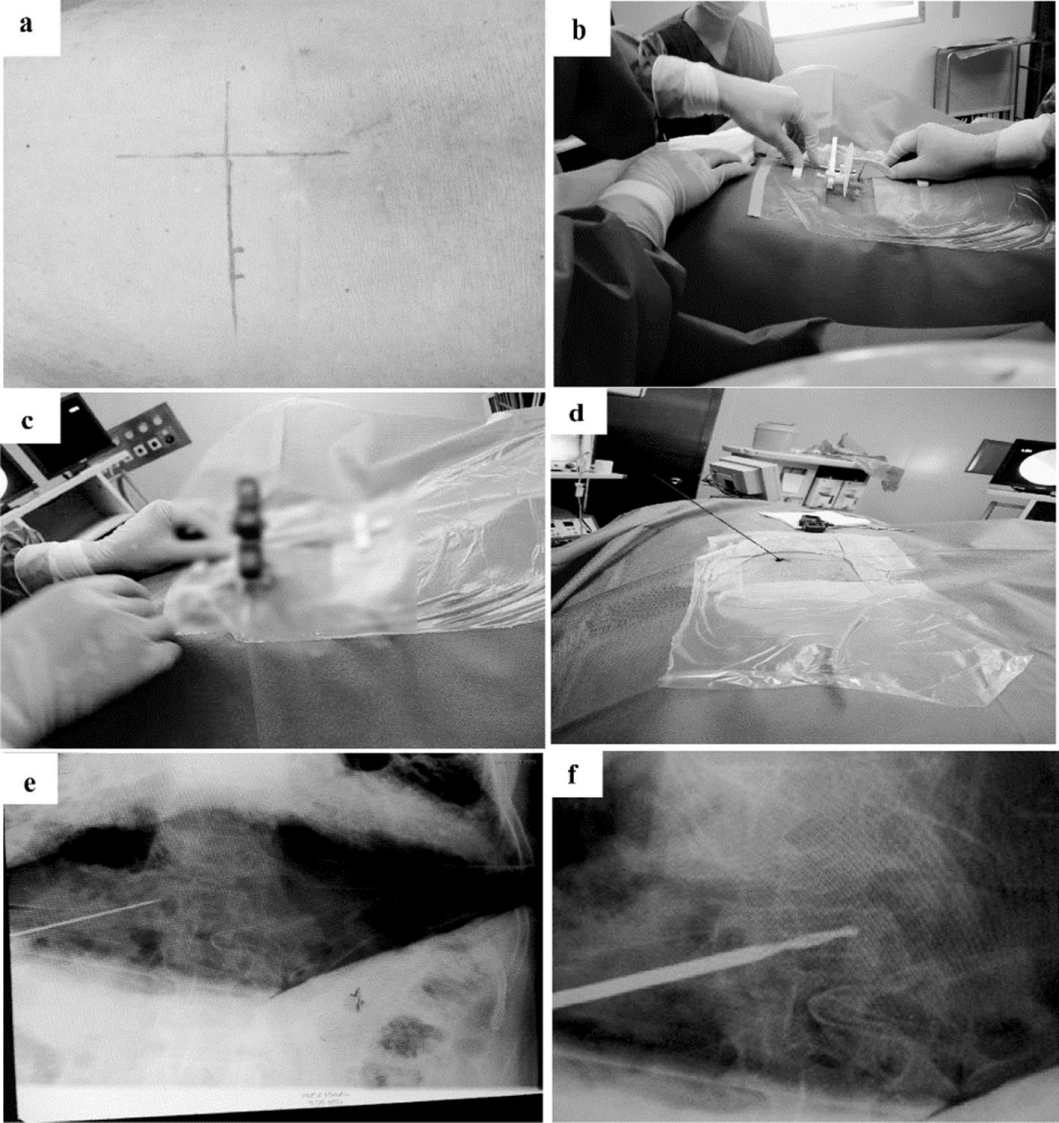
Percutaneous kyphoplasty by using the locator. Graph (**a**) shows that the patient was in the prone position. ML and HL lines, as well as the percutaneous puncture point “P” were marked according to the data acquired from the images preoperatively. Graph (**b**) illustrates that the locator was placed parallel to the ML line after adjusting for the positions of the hands on dials at the defined angles according to the preoperative measurement of extraversion angle α- and tail tilt angles β. Graphs (**c**) and (**d**) display that a cannula was placed in the locator, making the distal end of the cannula aligned with the puncture point “P”, and then, a guide needle was inserted into the cannula until the pedicle bone puncture point was planted into the pedicle. Graph (**e**) shows that the position of guide needle was confirmed by 2D fluoroscopy. Graph (**f**) depicts that a saccule was placed through the working channel and the fractured vertebral body was expanded with an appropriate pressure. Then, bone cement was injected into fractured vertebrae to stabilize the spine and relieve pain.

In Jinzhou Central Hospital, from May to December 2018, the locator was used in 9 cases of spinal fracture, 2 cases of spinal fusion, and 15 cases of PVP, with placement of 68 surgical needles. The data of before and after utilization of the locator were compared, including application of 69 surgical needles in 9 cases of spinal fracture, 2 cases of spinal fusion, and 17 cases of PVP. In the initial stage of using the locator, due to the substandard mark and a huge error in the locator’s position, the location of the guide needles should be readjusted in individual cases. Finally, all guide needles were accurately placed into the pedicles. As shown in Table 1, using the locator significantly improved the surgical efficiency than conventional freehand technique, and reduced the risk of X-ray exposure (P < 0.001, Table 1). The application of the proposed locator in some cases is shown in Figs. 3, 4.

**Table 1:** Comparison between the efficacy of the locator technique and the conventional freehand technique in MISS.

Characteristics	Locator technique n = 26	Conventional freehand technique n = 27	P*
The number of needles	68	69	–
**Gender, n (%)**			
Male	12 (46.15)	14 (51.85)	–
Female	14 (53.85)	13 (48.15)	–
Age, years (range)	64.42 ± 11.16 (39–85)	60.48 ± 13.12 (29–81)	–
BMI, kg/m^2^	24.71 ± 3.54	23.45 ± 2.55	–
Time required for insertion of needles per patient, min	3.04 ± 1.54	23.63 ± 12.03	< 0.001
The number of X-rays per patient	1.42 ± 0.58	5.37 ± 2.58	< 0.001

*P-value was assessed by comparing differences between the two groups via the Student’s t-test; *BMI* body mass index; Time required for placement of needles was the period from the time when the (first) guide needle started to penetrate the skin from point P on skin to the time when standard X-ray fluoroscopy showed that every the needle tip was at the midpoint of the pedicle’s lateral edge, and meanwhile, the X-ray lateral fluoroscopy showed that every guide needle was on the extension line of the pedicle’s midline; The number of X-rays indicated the frequency of taking X-ray fluoroscopy to check the position of guide needles during a surgery.

## Discussion

For decades, MISS has been well developed, due to advances and innovations of surgical instruments and techniques. MISS refers to any procedure that is less invasive than open surgery. Screws were originally placed via the conventional freehand technique and 2D fluoroscopy, increasing the risks of misplacement of screws and exposure to high levels of radiation^6^. Although the application of robotic-assisted surgery has significantly increased the accuracy of percutaneous pedicle screw placement and reduced the risk of radiation exposure^7^, the costly nature of robotic-assisted surgery has restricted its popularization in different hospitals. Moreover, robotic-assisted surgery may fail in certain unexpected circumstances.

In the current study, the structure of the percutaneous multi-function pedicle locator was simple. It was a sterile disposable medical device, and it was only used outside the patient's body, which was cost-effective and extremely safe. The design of the locator was based on the position of the pedicles suggested by the routine preoperative CT and X-ray fluoroscopic images of a patient. Several lines, two distances and two angles were used ingeniously to determine the puncture position on skin and to insert it into a pedicle of a Kirschner wire from the patient's body surface, hindering the complication of surgical procedures. Results of the current study suggested that the application of the percutaneous multi-function pedicle locator in MISS could greatly reduce the difficulty of placing surgical needles and increase accuracy of surgery. Different from the conventional freehand technique in MISS, it was able to allow confirming the placement of all the Kirschner wires with X-ray fluoroscopy at one time intraoperatively, remarkably shortening the operation time, mitigating harmful intra-operative radiation exposure for the patient, surgeons, and ancillary personnel of operating room. Additionally, its cost-effectiveness feature compared with robotic-assisted surgery is noteworthy. Therefore, it is convenient to carry out MISS using the proposed locator in various hospitals. Moreover, it could shorten the learning curve for young orthopedists to precisely locate pedicles in MISS, compared with the conventional freehand technique without robotic-assisted surgery. To our knowledge, the percutaneous pedicle screw placement with conventional freehand technique must be carried out by experienced surgeons, because it does not allow for gross visualization and tactile feeling.

In addition, the proposed locator could be applicable to intervertebral foramen microscopic surgery, however, the surgical procedures are slightly different.

In order to ensure the accuracy of pedicle positioning by using the proposed locator in MISS, several important notes were presented (refer to the section of “Important notes”). In the locator-assisted surgery, it was found that accurate positioning of the locator was the most important challenge. Because the back body surface of the patient on the operating bed was mainly unleveled, it was necessary to adjust the level of the locator with the assistance of a level instrument. In the same way, when marking the HL line on the body surface, a level instrument was also needed to ensure horizontal direction of the HL line. The disadvantage of the proposed locator was that there was no level instrument in the structure of locator, justifying why an additional level instrument was required to ensure the accuracy of a reference plane to the locator. Therefore, we will design a new locator with a level instrument to make the next surgeries easier and more convenient.

In fact, as long as lines, angles, and distances required for positioning of pedicle and the position of the proposed locator were accurate, a Kirschner wire could be placed accurately into the pedicle. For surgeons, their 3D imagination capability should be acceptable, and they should be familiar with the anatomy of the spine. In case of appearance of a deviation, they should timely adjust it appropriately using fluoroscopy.

In brief, the percutaneous multi-function pedicle locator has been proven to achieve the original goal of design, enabling orthopedic surgeons to quickly and safely complete personalized 3D positioning of the pedicle in MISS without computer-assisted navigation. Thus, orthopedic surgeons can perform MISS using the proposed locator when expensive computer-assisted navigation is absent.
